# Supplementary material for: The Genomic HyperBrowser: inferential genomics at the sequence level
Source: Genome Biol. 2010 Dec 23;11(12):R121. doi: 10.1186/gb-2010-11-12-r121 (PMC3046481; doi:10.1186/gb-2010-11-12-r121)
Supplement: Additional file 1 — Supplementary material. Miscellaneous supplementary material: gene coverage example. On the importance of realistic null models. On mathematics of genomic tracks. On system architecture. On Exon DNA melting example. Supplementary figures and tables. [file gb-2010-11-12-r121-S1.doc]

**Supplementary material for**

***Inferential genomics at the sequence level***

*Sandve GK, Gundersen S, Rydbeck H, Glad I, Holden L, Holden M, Liestøl K, Clancy T, Ferkingstad E, Johansen M, Nygaard V, Tøstesen E, Frigessi A, and Hovig E.*

1. Gene coverage example

How much of the human genome is covered with what may be defined as genes? A number of different gene definitions exist, depending on algorithmic and authoritative source. CCDS provides high confidence gene annotations from two manually curated sources, RefSeq and Hinxton (a union of Ensembl and Vega ). A third source of manual annotation is the H-Invitational (H-Inv) track . Taking CCDS as the reference definition (type:US), we find that 26% of the genome is covered by genes, ranging from 3% to 38% for chromosomes Y and 17, respectively. Refseq, Hinxton or H-Inv give global coverages of 35%, 43%, and 44%, respectively. Figure A shows the coverage of genes locally along the genome at cytoband resolution, according to each of these definitions. Note that the definition of a gene is here treated naively, in that we treat a gene as a segment from start of exon 1 to last exon inclusive, regardless of splicing variants, etc., as we here use it as a simple example only.


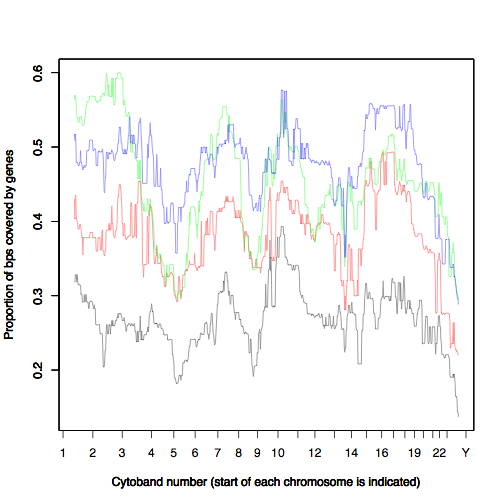


Figure A. The proportion of base pairs covered by genes along the genome (cytoband resolution) according to each of the gene definitions CCDS (black), Refseq (red), Hinxton (green), and H-Inv (blue). The curves have been median smoothed using a moving window with window size 51.

Gene length

To better understand the relation between different gene definitions, we describe the overlap between the reference definition CCDS and the other gene definitions as a function of gene length. For a pairs of gene definitions, a gene is included in the analysis if it occurs in at least one of the two definitions. Genes of one definition that overlap with more than one gene in the other definition are excluded from the analysis. For each gene the ratio of the number of base pairs covered by both gene definitions, and the number of base pairs covered by at least one of the two gene definitions is then computed. Figure B shows that the ratio of overlap between the two definitions increases when the gene length increases. Refseq has higher ratio of overlap and H-Inv has lower ratio of overlap for all gene lengths. The figure was made in R without any additional calculations, using output from the Genomic HyperBrowser.

Figure B. *Each of the gene definitions Refseq (red), Hinxton (green), and H-Inv (blue), are compared to CCDS. For each gene, the ratio of the number of base pairs covered by both gene definitions, and the number of base pairs covered by at least one of the two gene definitions, is plotted against gene length. Only genes with gene lengths larger than 1000 bp, according to CCDS, are included. The curves have been median smoothed, such that the smoothed ratio for gene length len is the median of the ratios that have lengths in the interval [len/2, len∙2].*

Quantification of gene overlap

The different gene definitions overlap more than expected if they were independent of each other. The hypothesis of independence between the reference definition CCDC and the other gene definitions is rejected in 96-98% of the cytobands where there are genes. Therefore, we want to describe how significant the overlap is when we take into consideration the number and length of the genes in each cytoband.

It is possible to make a confidence interval for the relative overlap between the two gene definitions in each cytoband. Define the variable Z for each cytoband as the number of base pairs inside both gene definitions. If the two gene definitions are independent of each other, then the expected value of Z is the product of the number of base pairs and the coverage ratio of the two gene definitions. If the genes are independent and at least one of the gene definitions only consists of many short genes, then the variance of Z is small. However, if both gene definitions are dominated by a few large genes, then the variance of Z is large since Z depends heavily on whether these few large genes overlap. We assume Z is normally distributed and estimate the variance of Z, σ^2^, assuming the two gene definitions are independent in a model where we permute the order of the genes and the intervals between the genes. Since the variance of Z mainly depends on the size distribution, it is natural to assume the same variance also in a model where the two gene definitions are dependent. The confidence interval for Z for the two gene definitions is then set equal to (z- c σ, z + c σ ) = (z_1_, z_2_) where z is the observed overlap between the two gene definitions in the cytoband and c=1.645 for a 90% confidence interval. The ratio of the overlap r is defined as r=z/(v_1_+ v_2_ –z) where v_1_ and v_2_ are the number of base pairs inside segments in the two gene definitions. The confidence interval for r is (z_1_/(v_1_+ v_2_ –z_1_), z_2_/(v_1_+ v_2_ –z_2_)). The confidence intervals for the relative overlap between the CCDC and Refseq gene definitions in the cytobands of chromosome 1 are illustrated in Figure C. The figure also shows the p-value and FDR value for rejection of the hypothesis that the two gene definitions are independent of each other. The figure shows that the confidence interval for the overlap is about (0.6,1) in most cytobands. The figure is made in R, without any additional calculations, using output from the Genomic HyperBrowser.

Figure C. *The figure shows the relative overlap in the independent distribution in red, the observed relative overlap of the gene segments in the CCDC and Refseq definitions in black, and a confidence interval for this overlap in the 63 cytobands in chromosome 1(shown as a black dashed line). Notice that there are three cytobands without any gene segment and three cytobands where the segments in the two gene definitions tend to be almost independent. In the remaining cytobands the observed relative overlap is much larger than in the independent model. The FDR value is shown in blue. It is close to zero in most cytobands, but defined to equal to1 in the cytobands without any genes. The p-value is in this example almost identical with the FDR and the curve is not possible to distinguish from the FDR curve.*

2. Supplementary material on the importance of realistic null models

Realistic assumptions on the properties of individual tracks are essential when studying the relation between tracks by significance testing. In our approach, we use the concepts of preservation and randomization in a generic scheme for controlling track assumptions. The importance of precise assumptions is easily seen by tests on simulated tracks, where we have full control of how tracks have been generated. We here show an example where failing to preserve an essential characteristic of one track leads to false positive discoveries on the relation between this and another track.

We generated a track A of points (UP) with a controlled degree of clumping, as described in Supplementary note on simulation. The points were generated with X and Y as mean distance between points within/between clusters respectively, and with probability Z of starting a new cluster (as opposed to continuing the current cluster). We also generated a track B of segments, with mean segment length given by a normal distribution with mean X, and distance between segments given by a geometric distribution with mean Y. We then asked the question whether points (of track A) are located preferentially inside segments (of track B), asking this same question for each 1 Mbp window of chr 1, i.e. in 248 bins. The null hypothesis is that points are located with same probability inside and outside segments (which we know is true, as track A and track B have been generated independently). We use MC-based significance evaluation, using count of points inside segments as test statistic, and randomizing positions of track A points in the null distribution. We use false discovery rate to account for multiple testing, using a FDR rate of 10%.

If we randomize track A points uniformly within each bin (only preserve number of points), we get around 30 false positive rejections of H0 among the 248 bins tested. This is because the clumping of points leads to a bigger variance in the number of points falling inside segments, giving a U-shaped p-value distribution, and thus leading to rejections even after FDR correction. If we in addition to preserving number of points also preserve the set of inter-segment distances, the same significance evaluation procedure gives no rejections of H0 (i.e. no false positives, as there are only true negatives). This is because preserving inter-segment distances preserves the essential characteristic of track A points for this question, reproducing the increased variance of number of points falling inside segments in the null distributions.

3. Supplementary material on mathematics of genomic tracks

Bins

Several binning strategies are available, including equally sized bins and bins built on or around genetic structure (genes, cytobands, etc.). Bins are decided before the analysis is carried out; however, if in a bin there are no or very few elements (points, segments, function values), then the statistical analysis lacks power, and the test will not be performed.

Genomic types

Points have no dimension, only a location. Questions related to points investigate their actual locations in comparison with the elements of the other track. If points are marked, then these marks might influence the location of points, and the way these interact with elements of the other track in the comparison.

Segments, as being intervals, have a location and a length. Questions related to their location only, can be easier answered by approximating each segment with a point (mid, left end, right end), and followed by comparison of this point with the elements in the other track. Questions related to the amount of genome covered by the segment, in addition to their location, require working with segments. For marked segments, the mark may have affected the length and/or location, and can interact with the elements of the other track in the comparison.

Functions have a domain of definition, which might be an interrupted part of the genome, or can be a set of intervals. The function takes a value on each base pair of its domain. This can be real, discrete or categorical. Questions investigate how the values of the function along its domain compare to the elements of the other track. For some questions, only level sets, corresponding to intervals of function value, are relevant, and these can be represented by segments. More generally, functions can be projected into (marked) segments in various ways.

Information reduction

An investigation may only need to use parts of the information contained in a track. For instance, if a study requires only the count of genes, then the length of the genes, *i.e.*segments, are unimportant. Thus, a track of genes can in this case be treated as unmarked points (UP), even though it is originally represented as unmarked segments (US). Such study–specific reduction of information is guided by the system, as it may require a choice for the approximation: which point should stand for the whole segment, the start-point, the middle point or the end–point of each gene?

Statistical comparison of tracks

Here are some examples of standard studies, in the language of geometry, for some pairwise comparisons. All questions are expressed in their global form, but they can easily be rephrased in the context of local analyses (“Where in the genome…?”). What is expected by chance is modelled by the null hypothesis.

- (UP,UP) The typical questions are formalised as counting points which have specific geometric properties. Examples: Are the point of track 1 more frequent than the point of track 2, more than expected by chance? Are the points of track 1 closer to the points of track 2, more than expected by chance? Are the points of track 1 accumulating to the left of the points of track 2, more than expected by chance?
- (US,US) Computing specific geometric properties of segments. Examples: Are the segments of track 1 overlapping more the segments of track 2, more than expected by chance? Are the segments of track 1 covered by the segments of track 2, more than expected by chance?
- (F,F) Computing specific analytic properties of and relations between the two functions. Examples: Are the two functions positively dependent, i.e. are high (or low) for the same base pairs, more than expected by chance? Do the two functions have similar derivatives, more than expected by chance? Do the two functions have peaks (spikes) around the same locations, more than expected by chance?
- (UP,US) Counting points within or outside the segments. Examples: Are the points of track 1 falling inside (or in the vicinity of) the segments more than outside the segments, more than expected by chance? Do they distribute uniformly within the segments?
- (UP,F) Computing specific analytic properties of the function in correspondence with the points. Examples: Is the function higher around the points of track 1, than what is expected by chance? Does the function have peaks (or high derivative) around the points of track 1, more than expected by chance?
- (US,F) Computing specific analytic properties of the function in correspondence with the segments. Examples: Is the function higher within the segments than outside, more than expected by chance?
- (MP,US) Computing specific geometric properties relating points and segments, depending on the marks. Example: Are the segments longer, when they contain more points with a certain mark, more than what expected by chance?
- (UP, MS) Computing geometric properties of the relation between points and segments, depending on marks. Example: Are points falling with differing frequency in segments depending on the mark value of segments?
- (MS,F) Computing specific analytic properties of the function in correspondence with the segments and depending on their marks. Example: Is the function higher in segments with a certain mark, than in segments with other marks, more than expected by chance?

The comparison can be alternatively expressed as "more" or "less" or "differently" etc, with implications on the way the test is sided. Notice that some questions are not symmetric. For each question, a test statistics T(T1,T2) is computed, which quantifies the comparison, depending on the elements in the two tracks. For example, if (T1=US,T2=US) and the question is "Are the segments of track 1 overlapping more than expected by chance the segments of track 2? ", then the statistics T must measure the length of overlap. It must also take into consideration the overall length covered by segments in the two tracks. The documentation of all implemented tests are provided in a separate note.

Given two tracks (T1,T2) and a test statistics of interests T, a null hypothesis is assigned by deciding the preservation rule and the randomisation scheme, for each track. The biologist needs to decide for each biological track which preservation is biologically reasonable and what randomness is best describing "nature".

More examples of preservation rules: For marked points, MP, preservation acts also on the marks. Here are some examples to illustrate the possibilities:

- Preserve the points of the track, but randomise the marks, possibly keeping the observed frequency of marks.
- Preserve the points of the track and permute the marks.
- Preserve the number of points, with their marks, but randomised the locations

Preservation of functions is more analytical:

- Preserve the level of smoothness.
- Preserve monotonicities (level sets).
- Preserve the strength of autocorrelation.
- Preserve the sign of the derivative.
- Preserve the location of peaks.

Preservation leads to conditional p-values, given preservation and randomisation rules. P-values are not ordered even if the preservations rules are so. This is in analogy to tests for two-by-two contingency tables, where row or column totals can be preserved - or not -, though p-values are not decreasing.

Intensity curves

Intensity curves, varying over the genome, are useful for biologically guided randomisation via the non-homogeneous Poisson process on the line (where basepairs take the role of time). The intensity λ_0_(b) describes how "nature" could have randomised the elements, say points, now observed as in the actual genomic track.

Intensity curves can be used to avoid randomisation of elements into specific areas of the genome, defining λ_0_(b)=0 on those areas. A third track can sometimes be used as intensity curve; for example, genes tend to appear where the GC content is high. The track GC content (F) can be used as intensity curve when randomizing genes. These would then appear in new positions, but still where GC tends to be high with larger probability.

Intensity curves can be used to adjust the study with respect to a third confounding track. Consider two tracks, which have a strong association in a chromosome under study. Assume that it is known that the elements in both tracks tend to appear in correlation with a third track. Therefore the direct comparison of the two tracks would get a rather expected positive answer. It is more interesting to study the relation between the two tracks, adjusting for the third track. We ask the question "Do the elements of the two tracks show positive association, more than what expected by the fact that both are associated to the third track?" To answer this question, we use the third track as intensity λ_0_(b) for generating the elements of the first track under the null. In this way, all simulated Monte Carlo configurations would adhere to the third track. Small p-values would indicate that the association between the two tracks is significantly higher (or lower) than what expected by their joint dependency on third track. A conclusion is then that there must be other phenomena that act on the association, in addition to the third track. This further mechanism interferes (or interacts) with the third track, to form unexpected levels of correspondence (or dissociation) between the two basic tracks. Intensity curves can take care of a known "confounding" effect represented by a third track. As an example, consider the relation between DNA melting and exon locations, which we know are both related to GC content. We wish to test if there is an additional association between melting (T1) and exons (T2), beyond the influence of GC. Let GC(b) be the density of GC content, acting as confounder track. First we determine the function s, which makes s(GC(b)) the best approximation of λ2(b), the estimated density of the exon track T2, by non-parametric Poisson regression function estimation (via spline functions). Under the null hypothesis, we randomize the points of T2 using λ0(b) = s(GC(b)).

Any track that is a function can be used as intensity curve. However, it might be useful to smooth the track first, or to make other preliminary operations. One possibility is to approximate the track with a piecewise constant function, over larger regions of the region under study. To represent forms of interaction, two intensities can be summed λ_1_(b) + λ_2_(b) to a new intensity, multiplied λ_1_(b) · λ_2_(b) or composed f(λ_1_(b), λ_2_(b)) through any other function f.

An interesting special case is when λ_0_(b) depends on the actual data (say points) of the track which is randomised. For example, if we are randomising points, then λ_0_(b) can be the empirical smoothed density of the observed points. This intensity curve would be high on areas where there are many points, and would fall to almost zero where there are few or no points. Local kernel estimator could be used, with appropriate smoothing.

In one of the biological examples, we use a confounding track, as mentioned in “methods section” of the paper. We give more details here on our implementation. We discretize the confounder tracks, by its step function approximation ζ_i_(b), on k chosen levels sets, q_1_,…,q_k_. Let the indicator function I_j_(b)=1 if the confounder track is at level q_i_ for position b, I_j_(b)=0 otherwise. Consider the track to be randomised (exon in the example); let I'(b)=1 if an event (e.g. an exon start) occurs at position b, I'(b)=0 otherwise. Then, for a bp b which is in the j’th level set, the intensity is given by

λ_0_(b) = ( Σ I_j_(b) I'(b) ) / Σ I_j_(b). This approach can be easily generalized to more confounder tracks.

Monte Carlo Test

When the exact calculation of the p-value is not possible, nor asymptotic arguments can be applied, standard or sequential Monte Carlo testing is performed.

Sequential Monte Carlo testing (10) is not a new idea, but rarely applied in genomics, despite its clear advantages. The method continues to sample until the sampled test statistics T is w times larger (or smaller, depending on side of the test) than the observed value of the same test statistics. Our choice for w is 20. Also, we fix a maximum number of samples. Sequential MC produces p-values that can be adjusted by FDR.

4. Supplementary material on system architecture

Extendibility

The Genomic HyperBrowser project is highly extendable at several levels. First, the analysis web-interface allows the built-in collection of annotation tracks to be easily complemented by any user-supplied track, and the built-in analyses options can be complemented by user-defined R code for the core analysis computation. Note that at our website, analysis based on user-defined R code is currently restricted to trusted users.

Second, the full system is available as modularized open-source code that can be downloaded to apply local customizations and extensions.

Third, our structured view of statistical genome analysis invites new questions on relations between annotation tracks to be proposed, additional assumptions to be considered for existing questions, and improved solutions to be devised.

Real runs to determine relevance

In a broad and complex system like the Genomic HyperBrowser it is for the user experience necessary to only present valid and possibly relevant options. At the same time, the complexity would grow out of control if one were trying to create and maintain explicit mappings of relevant options at each stage and possible state during analysis specification. A solution is to build in dynamic inference of relevant options throughout the system through a general mechanism for inferring relevance.

Two basic principles lie behind the architecture of the relevance inference mechanism: 1) At each stage the set of valid options should be determined by a procedure as similar as possible to a real run, and 2) restrictions should be specified close to the entities that are restricted (that is, validity of e.g. an analysis should be inferred from variables or code that are part of the analysis itself, not specified in an external relevance mapping code.) During track selection, the set of available options is inferred from the set of tracks found on disk. After tracks have been selected, the set of available analyses are determined by performing a minimal run of each analysis, presenting to the user only those analyses that run successfully, that is, those that are compatible with the selected tracks. Finally, the parameter options presented to the user are compiled from a custom parameter specification for the selected analysis.

Splitting data and avoiding redundancy

As the system should support a large and growing number of analyses, development of new analyses should be easy and efficient. This is supported in three main ways: 1) Shared intermediate computations, including the reading of track data from disk, are ubiquitously reused within a single run, so intermediate results can during development be requested without concern for computation redundancy between the requested intermediate statistics. 2) Computations on large regions are ubiquitously split to computations on smaller bins as long as code has been defined for computing the statistic from its value in sub-bins.

The general mechanism that ubiquitously splits data and avoids redundancy are based on a relatively simple architecture of the code that defines statistical computations. Each statistic defines a tree where the root represents the final result, where the leaves represent track data, and where internal nodes represent reusable intermediate statistics. The code for computing a statistic may request direct access to track data, request intermediate statistics, or request the same statistic computed for many small bins. In the first two cases, code must be provided to compute the statistic of interest based on track data and/or intermediate statistics, while in the last case code must be provided for combining the value of the statistic in each of many small regions to a statistic representing the full region of interest. All computations of statistics are by a general mechanism split to smaller bins of standardized size when possible, and the results of any statistical computation in a standardized bin is stored for later reuse.

Interoperability with Galaxy

Galaxy is a powerful platform for genome analysis that supports creating a web portal that handles users, running of tools, and organization of results. It also includes several useful tools for modifying files containing genomic data. Galaxy’s strengths as a portal and for modifying genomic data, means that it complements the analytical focus of the Genomic HyperBrowser very well.

Galaxy includes functionality for adding custom tools, and provides a simple mechanism that uses a custom XML-based format for defining the GUI of such tools. We have added several small tools in our local Galaxy installation through this mechanism. This mechanism is, however, focused on relatively small and independent tools. The Genomic HyperBrowser is a large system with its own integrated databases, and thus needs to run as a stand-alone application. Also, the highly dynamic nature of the system GUI posed more advanced requirements than is currently supported through the Galaxy tool mechanism, meaning that the system could not be integrated through this mechanism. Instead, we have developed a stand-alone GUI by the use of standard web components (HTML and Javascript). The GUI is accessed through the Galaxy menu, and communicates directly with the Galaxy portal through Mako. The effect is seamless integration, and for the user the Genomic HyperBrowser appears like any other tool in the Galaxy portal.

Disk memoization

As discussed elsewhere, all intermediate (and final) computations are split to computations on smaller pre-defined bins when possible. Performing computations on a standard set of pre-defined bins (every contiguous 100k base pairs of each chromosome in our current implementation) allows reuse of (intermediate) results across analyses, even when different analyses regions have been specified (as long as there are overlapping bins between the analysis regions of the two analyses). When a (intermediate) statistic is computed for a bin contained in the pre-defined set, the result is stored to disk with a key consisting of the statistic, the tracks it is computed from, all parameter options, and an index for the pre-defined bin. If the same computation is requested at a later time, a corresponding key will be generated, and the result will be loaded from disk instead of being computed again. All the functionality related to disk memoization is completely contained within the general backbone for statistics, meaning that it need not be considered when developing new statistical modules.

Data representation and interoperability

Track data is today mainly available in text-based formats. Although these formats have advantages, especially related to interoperability of data, they do not allow highly efficient representation of track data (which is mostly numeric), and does not directly support efficient non-sequential access (that is, efficient retrieval of track data for a specified region of the genome).

To cope with these limitations, we have developed a new track data format, which allow more efficient representation and supports indexing of data with respect to genome position. Using a new format, however, comes at the cost of less interoperability. Thus, we have used a combined approach, with parallel storage of tracks in a standard text format and in the newly defined format. More precisely, all imports and exports of data use the text format, which forms the main definition of track data, and are thus referred to as original format. The newly defined format is treated as a pre-processed version of the data, which is then a (more efficient) mirroring of the main data (automatically updated to reflect any changes to original data). All analyses in our system access the pre-processed data.

The standard text-based formats for track data are of two types: tabular text files and xml-files. We have used tabular text files as our main representation of track data, as track data in this format is abundant, and the files are human-readable and can easily be modified by simple scripts such as Galaxy tools. More specifically, we currently use the bed-, bedgraph-, wig- and microarray-formats, as defined by the UCSC genome browser. It would be easy to add support for different XML-based formats if needed, as our only requirement is code for parsing and converting these files to the pre-processed format.

The newly defined format is based on vectors of start positions, end positions and marks, which are stored directly to disk. Currently, this is implemented by using the “memmap” structure of the numpy package. Only the vectors needed for a certain track type are stored, so that e.g. point tracks do not have a vector of end positions. These “memmaps” are read/written efficiently between vectors in memory and files on disk.

Architecture for Monte Carlo significance tests

We have developed a general architecture for Monte Carlo tests that consists of three components:

1. A randomizer component that decorates a track. This component appears like a standard track, and delivers data that are based on the real data, but randomized according to a specified stochastic model of the data in a null hypothesis. Different null hypotheses thus give rise to different algorithms for randomizing track data.

2. An evaluator component that computes a p-value along with several descriptive statistics, based on a test-statistic on the real data and a distribution of the test-statistic on randomized data.

3. A manager component that creates randomized tracks, that distributes tracks to a statistical module for computing the test-statistic on real and randomized track data, and that finally delivers the values of the test-statistic to the evaluator component.

The three components are independent, and are also independent of the statistical module used to compute the test-statistic. The same manager and evaluator component is used for all Monte Carlo runs, while any test-statistic module and randomizer component can be combined to achieve a desired significance test (including the desired null hypothesis). This means that to extend the Genomic HyperBrowser system with a new Monte Carlo test, only a definition line needs to be provided that specifies which test-statistic and which randomizers to use in the test.

5. Supplementary material on Exon DNA Melting example

There are two melting fork probability tracks P_L_(x) and P_R_(x) used in the analysis in the paper. P_L_(x) is the probability that a left-facing fork (also called a “01”-fork, i.e. an open base pair followed by a closed) exists at position x and at the temperature T_m_(x) at which base pair x is open or closed with probability 0.5. Likewise, P_R_(x) is the corresponding probability for a right-facing fork. Because melting fork probabilities as a function of temperature typically attain their maximum close to the local melting temperature, the tracks P_L_(x) and P_R_(x) capture all melting forks existing at any temperature .

The GC-based function tracks, L(x) and R(x), were created using a moving window approach as follows: $L(x)=\sum_{i=0}^{100} i\cdot GC(x-100+i)$ is the GC content in the 100 bps flanking region to the left of position x summed with a triangle-shaped weight function (where the indicator GC is one if G or C, zero otherwise.) Similarly for the right side: $R(x)=\sum_{i=0}^{100} i\cdot GC(x+100-i)$. Both of the two intensity tracks created (for exon left and right boundaries, respectively) used the pair of L(x) and R(x) as controls.

6. Supplementary figures

Supplementary Figure 1: *The histone modification occupancy in 20 kb upstream and downstream regions of four groups of genes ranked according to their normalized expression value in CD4 T-cells . ~12000 genes have been divided into four groups of 3000 genes each. The "lowest" group contains some more genes (3541).*

Supplementary Figure 2. *The frequencies of H3K27me3 ChIP-chip segments from the cell lines MEFB1 and MEFF on mouse chromosome 17, divided into bins of size 5Mbps .*

7. Supplementary Tables

|  | **1 Kpbs Downstream** | **1 Kpbs Upstream** | **20 Kpbs Downstream** | **20 Kbps Upstream** |
| --- | --- | --- | --- | --- |
| **H3K9me1** | 0.257 (0.000) | 0.243 (0.000) | 0.332 (0.000) | 0.288 (0.000) |
| **H3K4me2** | 0.255 (0.000) | 0.237 (0.000) | 0.315 (0.000) | 0.291 (0.000) |
| **H4K20me1** | 0.323 (0.000) | 0.224 (0.000) | 0.361 (0.000) | 0.255 (0.000) |
| **H3K4me3** | 0.238 (0.000) | 0.218 (0.000) | 0.238 (0.000) | 0.249 (0.000) |
| **H3K4me1** | 0.233 (0.000) | 0.217 (0.000) | 0.315 (0.000) | 0.268 (0.000) |
| **H2BK5me1** | 0.275 (0.000) | 0.215 (0.000) | 0.361 (0.000) | 0.264 (0.000) |
| **H2AZ** | 0.174 (0.000) | 0.194 (0.000) | 0.151 (0.000) | 0.228 (0.000) |
| **H3K27me1** | 0.212 (0.000) | 0.172 (0.000) | 0.349 (0.000) | 0.276 (0.000) |
| **H3K36me3** | 0.181 (0.000) | 0.124 (0.000) | 0.377 (0.000) | 0.209 (0.000) |
| **H3K36me1** | 0.138 (0.000) | 0.104 (0.000) | 0.233 (0.000) | 0.187 (0.000) |
| **H3K79me1** | 0.103 (0.000) | 0.086 (0.000) | 0.227 (0.000) | 0.172 (0.000) |
| **H3R2me1** | 0.098 (0.000) | 0.084 (0.000) | 0.220 (0.000) | 0.164 (0.000) |
| **H3R2me2** | 0.050 (0.000) | 0.066 (0.000) | 0.178 (0.000) | 0.125 (0.000) |
| **H4R3me2** | 0.057 (0.000) | 0.051 (0.000) | 0.162 (0.000) | 0.119 (0.000) |
| **H3K9me2** | 0.011 (1.000) | 0.033 (0.000) | 0.052 (0.000) | 0.065 (0.000) |
| **H3K79me2** | 0.038 (0.000) | 0.022 (0.062) | 0.114 (0.000) | 0.087 (0.000) |
| **H3K27me2** | 0.000 (1.000) | 0.014 (1.000) | 0.031 (0.003) | 0.038 (0.000) |
| **H4K20me3** | -0.013 (1.000) | 0.005 (1.000) | 0.033 (0.001) | 0.043 (0.000) |
| **H3K9me3** | -0.009 (1.000) | -0.006 (1.000) | 0.007 (1.000) | -0.003 (1.000) |
| **H3K79me3** | -0.013 (1.000) | -0.006 (1.000) | 0.034 (0.000) | 0.028 (0.009) |
| **H3K27me3** | -0.094 (0.000) | -0.094 (0.000) | -0.115 (0.000) | -0.118 (0.000) |

Supplementary Table 1. *The correlation between histone modification occupancy and gene expression in regions around the TSS, sorted by correlation in 1 Kpbs upstream regions, with Bonferroni-corrected p-values in parenthesis. The correlation coefficient used is Kendall’s rank correlation coefficient.*

|  |  |  | **Chromosome arms** | | **Chromosome arms, split in 5 Mpbs bins** | |
| --- | --- | --- | --- | --- | --- | --- |
| **Genome** | **Track 1** | **Track 2** | **Significant bins** | **Invalid bins** | **Significant bins** | **Invalid bins** |
| hg18 | Barski | SINE | 0/43 | 5 | 0/531 | 80 |
| hg18 | Barski | LINE | 38/42 | 6 | 400/531 | 80 |
| mm8 | Mikkelsen | SINE | 19/21 | 1 | 0/475 | 52 |
| mm8 | Mikkelsen | LINE | 20/21 | 1 | 444/475 | 52 |
| mm8 | Pauler (MEFB1) | SINE | 1/1 | 0 | 0/19 | 0 |
| mm8 | Pauler (MEFB1) | LINE | 1/1 | 0 | 0/19 | 0 |
| mm8 | Pauler (MEFF) | SINE | 1/1 | 0 | 0/16 | 3 |
| mm8 | Pauler (MEFF) | LINE | 1/1 | 0 | 0/16 | 3 |

Supplementary table 2. *Overlap of different ChIP-based H3K27me3 segments as track 1 with SINEs and LINEs as track 2. The null model preserved all repeat segments exactly, preserved H3K27me3 segment and intersegment lengths, but randomized their positions. Two sets of hypothesis tests were carried out for each track combination, one on all chromosome arms as bins, and one with the chromosome arms split into 5 Mbps bins. The number of invalid bins, that is the bins where a p-value could not be calculated, is shown, as is the ratio of significant bins out of all valid bins. Note that hypothesis testing was carried out on both the human and the mouse genome.*

|  | Uniformity-based | | Intensity-based | |
| --- | --- | --- | --- | --- |
|  | Left | Right | Left | Right |
| chr1 | 0.0005 | 0.0005 | 0.487 | 0.556 |
| chr2 | 0.0005 | 0.0005 | 0.113 | 0.44 |
| chr3 | 0.0005 | 0.0005 | 0.16 | 0.259 |
| chr4 | 0.0005 | 0.0005 | 0.176 | 0.141 |
| chr5 | 0.0005 | 0.0005 | 0.263 | 0.0345 |
| chr6 | 0.0005 | 0.0005 | 0.14 | 0.358 |
| chr7 | 0.0005 | 0.0005 | 0.04 | 0.123 |
| chr8 | 0.0005 | 0.0005 | 0.304 | 0.421 |
| chr9 | 0.0005 | 0.0005 | 0.568 | 0.447 |
| chr10 | 0.0005 | 0.0005 | 0.222 | 0.143 |
| chr11 | 0.0005 | 0.0005 | 0.05 | 0.675 |
| chr12 | 0.0005 | 0.0005 | 0.574 | 0.247 |
| chr13 | 0.0005 | 0.0005 | 0.177 | 0.266 |
| chr14 | 0.0005 | 0.0005 | 0.519 | 0.183 |
| chr15 | 0.0005 | 0.0005 | 0.374 | 0.508 |
| chr16 | 0.0005 | 0.0005 | 0.191 | 0.225 |
| chrM | 0.0005 | 0.0005 | 0.0005 | 0.0005 |

Supplementary table 3. *P-values of the comparison of left (or right) melting fork peaks with the left (or right) exon ends as computed either with a uniform null hypothesis or an intensity-based null hypothesis that takes GC content variations into account [6].*
