# Supplementary material for: The Genomic HyperBrowser: inferential genomics at the sequence level
Source: Genome Biol. 2010 Dec 23;11(12):R121. doi: 10.1186/gb-2010-11-12-r121 (PMC3046481; doi:10.1186/gb-2010-11-12-r121)
Supplement: Additional file 2 — Statistical tests. Detailed description of the statistical tests implemented in the software system. [file gb-2010-11-12-r121-S2.pdf]

Statistical tests

This section describes the statistical tests in the computer system:

- UP-UP-Frequencies
- UP-UP-Distance Between Points
- US-US-Overlap
- US-US-Similar Segments
- UP-US-Located Inside
- UP-US-Located Nearby
- UP-US-Located Nonuniformly Inside
- UP-F-Higher Values At Locations
- US-F-Higher Value Inside
- F-F-Similarity
- MP-MP-Similar Marks In Nearby Points
- MP-MS-Similar Marks Of Points And Segments Where Points
- UP-MS-Located In Highly Marked Segments

At present there are some deviation between the implementation and the description below.

UP - UP, frequencies

Tracks

1. Track 1: unmarked points
2. Track 2: unmarked points

Question

Where is the relative frequency of points of track 1 different from the relative frequency of points of track 2, more than expected by chance?

Comment:

- This question is used to identify regions of the genome (or the part of it under analysis) where the two tracks are different, taking into consideration the unequal number of points in each track.
- "'more"' can be substituted with "'less"' or "'differently"'.
- For each region which is tagged as significant, we can identify cold spots, which are regions where Track 1 is significantly less frequent than Track 2, and hot spots, which are regions where Track 1 is significantly more frequent than Track 2.

Hypotheses tested

- In the local analysis, we test a null hypothesis in each bin and obtain a p-value for each bin.
- There is a possibility of global analysis, which compares the two frequencies over the whole area/genome.
- In each bin, we test the null hypothesis that the two tracks have the same relative frequency in that bin, against the alternative that the two relative frequencies are different. Two sided test.
- Only the total number of points in each track in the whole area of study is preserved.
- Randomisation could assume that blocks of bp's are switched, between neighbouring areas. This is not implemented currently.

Statistics

Our starting point is two sets of observations of positions of two genomic variables along the genome, n_1 positions of Track 1, and n_2 positions of Track 2 on the same interval (chromosome or genome) I. We consider these positions to be samples from two densities f and g on interval I, and want to test if the densities are unequal, that is, if the two sets of variables are positioned differently along the chromosome.

Globally, to test if two distributions are unequal, we can use global tests for distributions, ending up with one single (global) p-value for $H_0 : f = g$ against $H_1 : f \neq g$, for example Kolmogorov Smirnoff.

In the local analysis, we construct subintervals (bins) and we test if the relative numbers of points in a subinterval are different for the two tracks, and which track is under or over represented in that subinterval (hot and cold spots).

The most simple way to do the testing of proportions is described in the following: The interval I is subdivided into k non-overlapping bins of equal length, and in each bin we simply count the number of hits of Track 1 and of Track 2 in that bin.

For bin i, $i = 1, ..., k$ we obtain

$$\hat{p}_i = \text{fraction of Track 1 points in bin } i$$

and

$$\hat{q}_i = \text{fraction of Track 2 points in bin } i.$$

The underlying binomial probabilities are

$$p_i = P(\text{a Track 1 points is positioned in bin } i)$$

and

$$q_i = P(\text{a Track 2 point is positioned in bin } i).$$

We have to assume that the positions of points are independent.

To test if the underlying bin probabilities in bin i are diverse, we test

$$\begin{aligned} H_0 : p_i = q_i \quad & \text{against alternatives} & H_1 : p_i < q_i \\ & \text{or} & H_1 : p_i > q_i \\ & \text{or} & H_1 : p_i \neq q_i \end{aligned} \tag{1}$$

using some more sophisticated tests. Storer and Kim (JASA 1990) perform a comparison of seven such exact or approximate tests for the null-hypothesis above. The two most suitable of these are implemented in the Hyperbrowser.

Test Statistics

The simplest inference procedure is to use a z-statistic (needs at least a moderate number of points in the bin)

$$Z = \frac{\hat{p}_i - \hat{q}_i}{\sqrt{\frac{\hat{p}_i(1-\hat{p}_i)}{n_1} + \frac{\hat{q}_i(1-\hat{q}_i)}{n_2}}}$$

or, better, with pooled standard deviation, using that $p_i = q_i$ under H_0, giving

$$Z_{pooled} = \frac{\hat{p}_i - \hat{q}_i}{\sqrt{\frac{\hat{r}_i(1-\hat{r}_i)}{n_1} + \frac{\hat{r}_i(1-\hat{r}_i)}{n_2}}}$$

where $\hat{r}_i = (n_1\hat{p}_i + n_2\hat{q}_i)/(n_1 + n_2)$.

This latter approximate test is the 'winner' in Storer and Kim (1990) in the case of **unequal sample sizes** ($n_1 \neq n_2$), which we typically will have here. The comparison of

two proportions can alternatively be thought of as a 2x2 contingency table problem, where the z-test above will be identical to a χ^2-test. But the z-test has the advantage of allowing for one-sided alternatives while the χ^2-test only allows for a two-sided alternative hypothesis. Various continuity corrections etc. exist for the z-test above, but as none of these have proven superior and we need something fast, these are not worth implementing.

When the number of counts is too small for the approximate test above, we resort to Fisher's exact test. For bin i, we have a 2x2 table

	Track 1	Track 2
Inside bin i	$a = \hat{p}_i n_1$	$c = \hat{q}_i n_2$
Outside bin i	$b = n_1 - a$	$d = n_2 - c$

with the number of points inside and outside bin i for each track. Fisher's exact test tests if the probability of falling in bin i is the same for both tracks, that is, identical to the hypotheses above. The call in R is then fisher.test(data), where data is composed as matrix(c(a,b,c,d) nr=2), and the p-value is computed based on the hypergeometric distribution taking all possible configurations in the table into consideration. This call automatically calculates a two sided p-value, but it is possible to alter this.

There are also alternative versions of this test, where row and columns sums are not fixed, discussed f.ex. in Storer and Kim, but we conclude that these complications are not worth implementing, since the results are rather similar, the calculations even more demanding and Fisher's exact test is considered conservative.

If there are so few points in a bin that not even Fisher's exact test can be performed, no p-value will be calculated and the Hyperbrowser returns 'NA' for that bin.

UP - UP, distance between points

Tracks

1. Track 1: unmarked points
2. Track 2: unmarked points

Questions

Where in the genome are the points in track 1 closer to/further apart from points in track 2 than expected by chance?

Comment:

- We assume points in track 2 as fixed and want to find out whether points in track 1 are closer to or further apart from the closest point in track 2 than expected. The test may indicate that the two tracks are independent. The test is not symmetric in the two tracks.
- Significance is determined by means of p-values. Small p-values identify regions where the points in track 1 are closer to or further apart from the closest point in track 2 than expected. P-values are computed as explained below, where the null hypothesis is explained in detail.
- The p-values may be found by simulation or by an approximate calculation. It is necessary to specify a distribution of the unmarked points in track 1.

Bins

The genome (or the areas of the genome under study) are divided into small regions, called bins. The tests are performed in each bin.

Hypothesis tested

For each bin i we have the four different null hypotheses corresponding to each of the four alternative preservation rules given below:
$\mathbf{H}_0$: *Assume points in track 1 are independent of points in track 2*
with the following alternative hypotheses:
$\mathbf{H}_1$: *Points in track 1 are closer to points in track 2 than expected* or
$\mathbf{H}_2$: *Points in track 1 are further apart from points in track 2 than expected.*

Let $g(r)$ be the point in track 2 that is closest to the point r in track 1. Define the distance $d(r)$ as the distance between the position of r and the position of $g(r)$ (see Figure 1). Let $r_1, \ldots, r_n$ be the points in track 1 in bin i, and let $\hat{\mu} = \frac{1}{n}\sum_{j=1}^{n} d(r_j)$ be the mean distance between points the tracks 1 and it's nearest point in track 2. In all tests the points in track 2 will be considered as fixed, the points in track 1 as random and $\hat{\mu}$ will be used as test statistic.

The H_0 hypothesis is rejected for each bin i if: $\hat{\mu}_i > c_{\alpha,i}$ or $\hat{\mu}_i < d_{\alpha,i}$ or $c_{\alpha/2,i} < \hat{\mu}_i < d_{\alpha/2,i}$ corresponding to the average distance is significantly larger/smaller/different than expected. The critical values $c_{\alpha,i}$ and $d_{\alpha,i}$ are found by simulation and depend on the threshold α and the bin i.

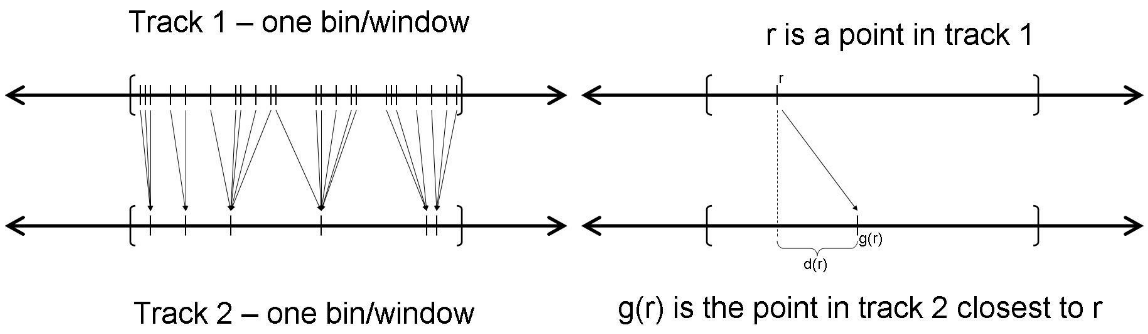

Figure 1: *Comparing positions in track 1 and 2.*

We may assume four different preservation rules for the distribution of points in track 1. These give different null distributions for $\hat{\mu}$ and hence different test results. In all four cases we use Monte-Carlo simulation for obtaining samples of track 1 under the null hypothesis. For each sample of track 1, the corresponding $\hat{\mu}$ is computed and the distribution of $\hat{\mu}$ under the null hypothesis is obtained. How to sample the points of track 1 under each of the four different preservation rules is described below.

Preservation rule 1: Preserve the number of points in the bin in track 1 Assume track 1 has n points. The locations of the n points are drawn independently and uniformly in the bin.

Preservation rule 2: Preserve the number of points and also the interpoint distances in the bin in track 1 The points in track 1 are sampled by permuting the interpoint distances of the original track 1.

Preservation rule 3: Preserve the distribution of the interpoint distances in the bin in track 1 The leftmost point might be drawn by drawing a distance d from the distribution D of the interpoint distances, and then draw the distance from the bin start to the first point from the uniform distribution U[0,d]. The next points in track 1are sampled one by one from left to right by drawing the interpoint distances from the distribution D. We stop drawing new points when the next point would have been placed outside the bin.

If a control track is available the four sampling procedure above might be extended as indicated in the note "Sampling MC-locations from the candidate track".

Approximation under preservation rule 1

For preservation rule 1 we may, alternatively, use an approximation for the null distribution of $\hat{\mu}$ as described below.

Assume that the number of points in the bin in track 1 is preserved. Let $D_1, \ldots, D_n$ be independently, identically distributed random variables for the distances of the points in track 1, $d(r_1), \ldots, d(r_n)$.

The locations for the n points in track 1 are independent. Let f be the prior on possible locations for one point. In the special case that f is uniform, we observe that the distribution of each of $D_1, \ldots, D_n$ is a mixture of non-overlapping uniform distributions i.e. the

distribution f_D is a piecewise constant distribution (Figure 2):

$$f_D(d) = \sum_{i=1}^{m} c_i \cdot U[b_{i-1}, b_i],$$

where m, a_i and b_i, $i = 1, \ldots m$, are as indicated in Figure 2, $b_0 = 0$ and c_i is the fraction of the bin that is covered by a_i segments. b_1 is the shortest half-distance, b_2 the next shortest etc. and $a_i = b_i - b_{i-1}$.

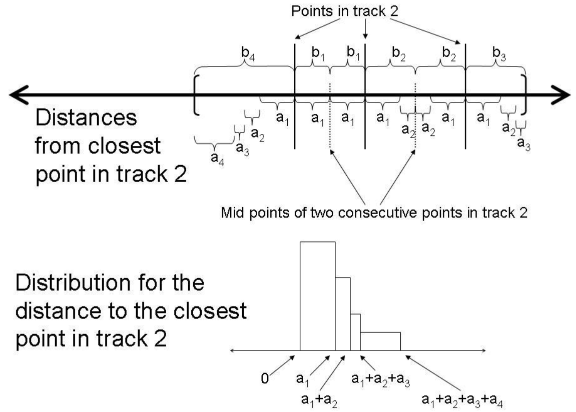

Figure 2: *Distribution of the distances of the points in track 1 for a bin with three points in track 2 assuming a uniform prior.*

When the prior on possible locations is not a uniform, the distribution f_D for the distance to the closest point in track 2 is obtained as follows: Let m be the largest possible distance and define a function $g : \{0, \ldots m\} \rightarrow R$ by $g(i) = \sum_{\text{location } l \text{ with } d(l)=i} f(l)$. Then $f_D(i) = \frac{g(i)}{\sum_{j=0}^{m} g(j)}$. Also in this case f_D is a piecewise constant distribution, but each interval with constant values is very short. To obtain longer intervals we might approximate $f_D(i)$ with another piecewise constant distribution, f.ex. by repeatedly merging some neighbour intervals with quite similar values into a new interval with constant value equal to the mean of the original values.

The null distribution for μ, the mean distance for the points in track 1, may be approximated by a piecewise constant density. The density may be found by adding one and one of the terms in the sum (this should be done in such a way that as few additions as possible are performed). When the number of points in track 1 is large, the null distribution for μ might be approximated by a normal distribution. Small p-values are obtained when $\hat{\mu}$ computed from the data occurs in the left/right/left or right tail of the null-distribution, corresponding to the tests of whether the average distance is significantly larger/smaller/different than expected.

US - US, overlap

Tracks

1. Track 1: unmarked segments

2. Track 2: unmarked segments

Questions

Where in the genome do the segments of track 1 intersect the segments of track 2, more than expected by chance?

Comment:

- This question is used to identify regions of the genome (or the part of it under analysis) where segments in the two tracks overlap more than expected.

- The global analysis answers the question "'Do the segments of track 1 overlap with the segments of track 2, more than expected by chance?"'

- "'More"' can be changed into "'less"' or "'differently"'.

- The p-values are computed exactly, asymptotically or found by simulation. This depends on the null hypothesis chosen. Simulation takes more computing time. It might be advisable to start with the hypothesis which preserve less, and require no simulation, to get a first impression.

- Not all the options described in this note are currently implemented.

Null Hypothesis and test statistics

We consider one bin. Consider the segments in track 1 in that bin. The elements that characterise this track are the segments, which are in a certain number l_1, of certain lengths each, and positioned in certain places of the bin. Between segments, there are also segments, here called intersegments. They also have a cardinality (which is one the three $l_1 - 1, l_1, l_1 + 1$) and a length and position. There are several levels of preservation of this structure, which are used to describe various null hypothesis: (i) Preserve all, exactly as is in the data; (ii) preserve the segments and the intervals between segments (inter-segments), in number and length but not their positions; (iii) preserve only the segments, in number and length, but not their positions; (iv) preserve the number of segments but not their length, nor position; (v) preserve only the number of base pairs in segments, not their position nor number, hence not the segments themselves. Because two different preservation rules can be decided for each of the two tracks, the test will often be not symmetric.

A statistics is defined that measures the overlap of the segments. There are several possibilities. One could use the segments as units, and just count how many segments in track 1 have an overlap with segments in track 2. In this case it makes no difference if the overlap is large in terms of basepairs (bp's), or just small. Instead, we will measure how many basepairs the overlap measures, and compute the probability of the observed overlap under the null hypothesis. Here is a precise mathematical definition of the statistics

Let $i = 1, 2, ..., n$ be indicating the n bp in the bin (or chromosome or whole genome). Let

$$X_i = 1 \text{ if bp } i \text{ is in a segment of track 1,} \tag{2}$$
$$X_i = 0 \text{ otherwise.} \tag{3}$$

And similarly for track 2:

$$Y_i = 1, \text{if bp } i \text{ is in a segment of track 2,} \tag{4}$$
$$X_i = 0 \text{ otherwise.} \tag{5}$$

Then

$$T = \sum_{i=1}^{n} X_i Y_i$$

is the total number of bp's (in the bin) which are within segments of both tracks. T/n is then the percentage of bp's covered by segments in both tracks. Sometimes it is more interesting to compute the percentage of bp's in the segments of track 1 which are covered also by segments in track 2. This is then

$$\frac{T}{\sum_{i=1}^{n} X_i}.$$

All these are possible test statistics. For some preservation rules and randomisations, the corresponding p-value can be computed exactly.

Null Hypothesis 1, very unequal preservation in the two tracks

The null hypothesis is given by:

1. Preserve all in track 2: the observed data.
2. In track 1, preserve only the expected number of bp which fall in a segment. That is the expected number of bp must be $\theta_1 = \frac{1}{n} \sum_{i=1}^{n} X_i$
3. In track 1, each bp is either inside or outside a segment with probability θ_1 independently of each others.

Note that this null hypothesis does not preserve anything of the segment stricture of track 1, except for the expected number of bp's covered by segments. It is possible to make an exact calculation for this simple null hypothesis:

$$P(T > k) = P(\sum_{i=1}^{n} X_i Y_i > k) = P(\sum_{i s.t. Y_i = 1} X_i > k).$$

Assume that $b_2 = \sum_{i=1}^{n} Y_i$ is the number of bp in track 2 covered by segments. The last sum is over b_2 terms. Under the null hypothesis point 3 above, the X_i's are iid, with $P(X_i = 1) = \theta_1$. So their sum is distributed according to a Binomial(b_2,θ_1). Hence

$$P(T > k) = \sum_{h=k+1}^{b_2} \binom{b_2}{h} \theta_1^h (1 - \theta_1)^{b_2 - h}$$

is the exact p-value.

It is possible to make an asymptotic approximation, to avoid computing these sums. Here we use that the binomial is approximated by a normal. More precisely, a Binomial(b_2,θ_1) random variable has approximately a normal distribution

$$N(b_2\theta_1, b_2\theta_1(1-\theta_1)).$$

Hence

$$P(T > k) \sim 1 - \Phi(\frac{k - b_2\,\theta_1}{\sqrt{b_2\,\theta_1(1-\theta_1))}}$$

asymptotically. We can use this approximation when

$$b_2\theta_1 > 5, \quad \text{and } b_2(1-\theta_1) > 5.$$

Null Hypothesis 2, more realistic

The null hypothesis is given by:

1. Preserve all in track 2: the observed data.
2. In track 1, preserve the segments but not their positions, nor the intersegments.
3. In track 1, each segment is positioned at random, independently of each others, but with no overlap. This is a random permutation.

Under this model, the statistics

$$T = \sum_{i=1}^{n} X_i Y_i$$

has a distribution cannot be computed exactly. [To explain why, first observe that

$$T = \sum_{i=1}^{n} X_i Y_i = \sum_{i,\,:\,Y_i=1} X_i,$$

as track 2 is fixed. The random variables X_i are not independent anymore. For example, say that $Y_7 = Y_8 = 1$: if $X_7 = 1$, then it means bp 7 is in a segment of track 1. As this segment will probably continue over bp 7, it is very likely that $X_8 = 1$, too. Hence dependence.] We can do asymptotics: It is possible to use a central limit theorem for sums of dependent variables. Under the assumption that the dependence is not too strong, then the limit is still normal, but the asymptotic variance is larger and more complicated to estimate. More precisely, if the X_i's is a mixing random process along the genome, then this is enough. Mixing means, that random variables far apart from one another are nearly independent. A formulation of the central limit theorem under strong mixing is given in (Billingsley 1995, Theorem 27.4). The asymptotic variance of T is

$$\sigma^2 = \mathrm{E}(X_1^2) + 2\sum_{k=1}^{\infty} \mathrm{E}(X_1 X_{1+k}).$$

One could now estimate from the data in track 1 the expectation $\mathrm{E}(X_1X_{1+k})$ as

$$\frac{1}{b_1} \sum_{i\,:\,Y_i=1, \text{ and } Y_{i+k}=1} X_i X_{i+k}$$

for several values of k, until this becomes small and can be ignored in the sum in σ^2. This is computationally intense, but feasible. There is also the possibility to assume a parametric model for $\mathrm{E}(X_1 X_{1+k})$, as a function that decays geometrically fast to zero in $k2$. In this case one needs to estimate the parameters of this decay function from the data in track 1.

There remains the possibility to estimate $P(T > k)$ under the null hypothesis by Monte Carlo. For this purpose, we need to produce random permutation of the segments. There are several algorithms to do this. We use this one: Preserving the lengths of the segments, means that we know the total length of the intersegments too. Then the algorithm starts with splitting the total intersegment lengths in $l_1 + 1$ parts (or l_1, that depends if the bin starts with a segment or with an intersegment in the data). We then take first a segment, then an intersegment, then a segment etc. until all are used. This gives a random permutation. Notice that this algorithm can easily be used also to sample from the null hypothesis that preserves also all intersegment lengths, as we would then simply sample from the bag of such intersegments, instead than generating a random partition of the total intersegment length.

Null Hypothesis 3, random permutations of segments and intersegments

The null hypothesis is given by:

1. Preserve all in track 2: the observed data.

2. In track 1, preserve the segments but not their positions, and the intersegments, but not their positions,

3. In track 1, each segment and intersegment is positioned at random, independently of each others, but with no overlap. A segment is followed by an intersegment. This is a random permutation.

This can be done by Monte Carlo, as explained in the simpler case when the intersegments are not preserved.

Null Hypothesis 4, for both tracks, random permutations of segments and intersegments

The null hypothesis is given by:

1. In track 1, preserve the segments but not their positions, and the intersegments, but not their positions,

2. In track 1, each segment and intersegment is positioned at random, independently of each others, but with no overlap. A segment is followed by an intersegment. This is a random permutation.

3. In track 2, assume the same as in track 1.

This can be done by Monte Carlo, as in the previous case, by sampling both tracks before computing the statistics T.

Null Hypothesis 5, very unrealistic in both tracks

The null hypothesis is given by:

1. In track 1, preserve only the expected number of bp which fall in a segment. That is the expected number of bp must be $\theta_1 = \frac{1}{n}\sum_{i=1}^n X_i$
2. In track 1, each bp is either in or outside a segment with probability θ_1 independently of each others.
3. In track 2, assume the same as in track 1.

This case can be done exactly, as we suggested in the analogous case when one of the track is fixed and in the other we just preserve the expected number of bp's within segments:

$$P(T > k) = P(\sum_{i=1}^{n} X_i Y_i > k) = P(\sum_{i=1}^{n} G_i > k),$$

where G_i are iid, equal to 1 with probability $\theta_1 \cdot \theta_2$, so that T is Binomial(n,$\theta_1 \cdot \theta_2$), with n number of bp's.

A different test statistics

Assume now we just count the number of segments which overlap, ignoring how large the overlap is in terms of bp's. In each given bin, we count how many segments of track 1 have a non-empty intersection with a segment (or many segments) of track 2. Let $Z_j = 1$ if segment j in track 1 has non-empty intersection with segment(s) of track 2, $Z_j = 0$ otherwise. Then

$$\frac{1}{l_1}\sum_{j=1}^{l_1} Z_j$$

is the percentage of segments in track 1 intersecting segments in track 2. Under various null hypothesis, it is possible to compute exact and asymptotic distributions for this statistics. Monte Carlo is also possible. The above statistics is natural if the segments of track 2 are preserved. It is possible to invert the role of the two tracks, and get a similar statistics.

US - US, similar segments

Tracks

1. Track 1: unmarked segments
2. Track 2: unmarked segments

Question

Where in the genome are the segments of track 1 similar to the segments of track 2 with more/less/different frequency than expected by chance?

Comment:

- This question is used to identify regions of the genome (or the part of it under analysis) where segments in the two tracks are very similar, i.e. almost overlapping. Similar is defined as follows: Let S_1 and S_2 be two segments in track 1 and track 2 respectively that overlap. Define S_3 as the union of S_1 and S_2 and $l(S)$ as the length of a segment S. That S_1 and S_2 are similar is defined as $l(S_1)/l(S_3) > \beta$ and $l(S_2)/l(S_3) > \beta$ for a constant β. The test is then based on the ratio of the segments in the bin that is very similar to a segment in the other track. The test is symmetric in the two tracks.
- Significance is determined by means of p-values. Small p-values identify regions where the segments in the two tracks overlap more/less/different than expected. P-values are computed as explained below, where the null hypothesis is explained in detail.
- The p-values are found by simulation. It is necessary to specify a distribution of the unmarked segments. We specify the following distribution. The user specifies bins or if used globally two endpoints. Then we assume the length of all segments and all intervals between segments including interval between first and last segment and corresponding end point as fixed. New realizations are simulated by permuting the order of the segments and the order of the intervals between segments in the two tracks.

Bins

The genome (or the areas of the genome under study) are divided into small regions, called bins. The tests are performed in each bin.

Hypothesis tested

Question, similar segments

- Hypothesis: The frequency of similar segments is not larger/smaller/different than expected.

  Note that this test depends on the threshold β defined above.

- Observator for bin i: K_i = number of segments that are similar with segment in the other track in bin i/ number of segments in track 1 and 2

- The p-value of the test is found from the distribution of K_i depending on the hypothesis and the distribution for the segments under the null hypothesis.

UP - US, located inside

Tracks

- Track 1: Unmarked points
- Track 2: Unmarked segments

Questions

Where in the genome is there more points of track 1 inside the segments of track 2 than expected by chance?

Comments:

- This question is used to identify regions of the genome (or the part of it under analysis) where points are over-represented inside the segments and where this over-representation is so strong that it would seldom happen by chance. Such over-representation would be a strong indication that points and segments do not occur independent of each other.
- In the analysis, the genome is divided into bins, and the tests are carried out for each bin
- ”more” may be changed into ”less” or ”either more or less”.

Hypothesis tested

The model valid under the null hypothesis is given by:

1. a preservation rule for each track,
2. a probability law on how the non-preserved elements are randomized. This rule implicitly imply independence of the positioning of the points and segments in the two tracks.

Consider the points in track 1 in one bin. A challenge in formulating models is that the structure of the interpoint distances may be crucial. If points (under the null hypothesis) are randomly distributed, or more precisely, conform to a Poisson process, then there is a simple solution to the testing problem (see Null hypothesis 1 below). However, there will probably often be more structure in the sequence of points: the points may occur more regularly than in a Poisson process, but probably more importantly, they may form clusters of points. Such clustering is very difficult to model. Thus, in the solutions presented below we either make the strong assumption of random positioning of the points (Null hypothesis 1) or we preserve the point positions in track 1 and base the tests on specific assumptions regarding the random segmental structure of track 2.

Let N be the total number of points in track 1 in the bin under consideration, and let T be the number falling within the segments defined by track 2.

Null Hypothesis 1, points in track 1 are randomly distributed

The model valid under the null hypothesis is:

1. The number of points in track 1 is preserved and the points are assumed uniformly distributed (typically arising from a simple Poisson process).

2. A fraction of the bp in track 2 equal to the observed one is included in segments.

Note that assumption 1 above is a very strong assumption on lack of structure for the points. The gain from this strong assumption is that we get a simple test and only need to make a very weak assumption for track 2.

The p-value is $P(T \geq k)$ where k is the observed value of T in the data. (If the question would be "less", we would use $\leq$; if "different" we would multiply times two.)

Let θ be the fraction of bp in track 2 that belongs to segments. Then

$$P(T \geq k) = \sum_{h=k}^{N} \binom{N}{h} \theta^h (1-\theta)^{N-h}$$

gives the exact p-value.

It is possible to make an asymptotic approximation, to avoid computing these sums. Here we use that the binomial is approximated by a normal. More precisely, a Binomial(n,θ) random variable has approximately a normal distribution

$$N(n\theta, n\theta(1-\theta)).$$

Hence we may approximate by

$$P(T \geq k) \sim 1 - \Phi(\frac{k - 0.5 - N\,\theta}{\sqrt{N\,\theta(1-\theta)}})$$

asymptotically. The 0.5 is a continuity correction, making the approximation better.

Null Hypothesis 2

The model under the null hypothesis is given by:

1. Track 1 is preserved as observed

2. In track 2, we preserve the segment lengths, but not the segment ordering or positions.

The test statistics remain the same as above; the number T among the N points that falls within a segment. However, now T has a distribution under the null hypothesis which we are not able to find exactly.

We thus compute p-values using Monte Carlo simulations. We do this by generating many new configurations of track 2 (in the bin we are working on). Each repetition has the same segments as the data (same collection of segment lengths), but now with random ordering of the segments within the bin and with random distances between the segments.

Preserving the lengths of the segments, means that we know the total length of the inter-segments too. If there are K segments, the algorithm starts by splitting the total intersegment lengths L into K parts (intersegments) by drawing K-1 points on [0, L]. A realization of track

2 is then obtained in a two step process. The reason for the two steps is that the borders of the bins represent a challenge in the implementation: using the trivial solutions, points in track 1 close to the segment border will either have larger or smaller probability of being included in a segment. Therefor, in the first step we make a sequence: the first intersegment, followed by a randomly drawn segment, then the next intersegment, the next randomly drawn segment and so on. Then we connect the borders of track 2 (if both borders are covered by segments, we still regard them as two segments). Finally, we randomly draw a starting position on the circle and use this as the starting point for the bin.

UP - US, distance between nearby points and segments

Tracks

1. Track 1: unmarked points
2. Track 2: unmarked segments

Question

Where in the genome are the points in track 1 closer to/further apart from the segments in track 2 than expected by chance?

Comment:

- The test is valid for all combinations of the alternative combinations of preservation and randomization of points in track 1 and segments in track 2. The test is not symmetric in the two tracks.
- Significance is determined by means of p-values. Small p-values identify regions where the points in track 1 are closer to or further apart from the closest segment in track 2 than expected. P-values are computed as explained below, where the null hypothesis is explained in detail.
- The p-values are found by simulation.

Bins

The genome (or the areas of the genome under study) are divided into small regions, called bins. The tests are performed in each bin.

Hypothesis tested

Hypothesis tested

For each bin i we have the null hypothesis:
$\mathbf{H}_0$: *The points in track 1 are independent of the segments in track 2.*
and the following alternative hypotheses:
$\mathbf{H}_1$: *Points in track 1 are closer to the segments in track 2 than expected* or
$\mathbf{H}_2$: *Points in track 1 are further apart from the segments in track 2 than expected.*

Define the distance d_i as the smallest distance between point i in track 1 and a segment in track 2 for $i = 1, 2, \cdots, n$. If the point i is inside a segment, then $d_i = 0$. We use the test statistics $X = \frac{1}{n}\sum_{i=1}^{n} d_i$. The distribution for this test statistics is not know and it is necessary with MC simulation in order to decide whether to reject the hypothesis.

UP - US, uniform positioning of points within segments

Tracks

1. Track 1: unmarked points
2. Track 2: unmarked segments

Questions

Q2-1 Where in the genome are the points in track 1 positioned more towards the borders of the segments in track 2 than expected by chance?

Q2-2 Where in the genome are the points in track 1 positioned more towards the middle of the segments in track 2 than expected by chance?

Q2-3 Where in the genome are the points in track 1 positioned more towards the left part of the segments in track 2 than expected by chance?

Q2-4 Where in the genome are the points in track 1 positioned more towards the right part of the segments in track 2 than expected by chance?

Q2-5 Where in the genome are the points in track 1 positioned more non-uniformly inside the segments in track 2 than expected by chance?

Comments:

- We assume that segments are fixed and regard points as random and independent.
- Significance is determined by means of p-values. For Q2-1, small p-values identify regions where the points in track 1 are closer to the borders of the segments in track 2 than expected. Similar for Q2-2, Q2-3, Q2-4 and Q2-5.

Bins

The genome (or the areas of the genome under study) are divided into small regions, called bins. The tests are performed in each bin.

Hypothesis tested

H_0: Points have a uniform distribution within segments.

Remarks

- If points are uniformly and independently distributed over segments, this will also be the case even if we rescale all segments to the same length.
- The tests described here are restricted to situations where such rescaling appears reasonable. This may not always be the case; biologists may for instance be interested in the distribution of the length in absolute terms from the start of the segments. This will, however, often be an estimation problem rather than a testing problem.

- If points are uniformly distributed, they are symmetrically distributed around the mean/median value. This may be used to construct tests.

Alternative hypotheses

$\mathbf{H}_1$: Points tend to be positioned towards the borders of the segments.

$\mathbf{H}_2$: Points tend to be positioned towards the middle of the segments.

$\mathbf{H}_3$: Points tend to be positioned towards the left part of the segments.

$\mathbf{H}_4$: Points tend to be positioned towards the right part of the segments.

$\mathbf{H}_5$: Points are unequally distributed within segments.

Testing against $\mathbf{H}_1$, $\mathbf{H}_2$, $\mathbf{H}_3$ and $\mathbf{H}_4$

When testing against H_1 and H_2, let d_i, $1 = 1, \ldots n$, be the relative position, but now scaled such that the value is -1 at both borders and 1 in the middle of the segment (and thus 0 halfway between the middle and the border).

When testing against H_3 and H_4, let d_i, $1 = 1 \ldots n$, be the relative position of points within segments scaled such that the value is -1 at the left end and 1 at the right end.

To test the first four hypotheses above, we may use the Wilcoxon sign-rank test. For n larger than 20-30, we may also use the t-test, which is markedly less time-consuming.

The Wilcoxon test is done in the following way: Rank the d_i without regard to sign; with 1 assigned to the observation closest to 0 (any zeros are neglected). Then compute $W+$ and $W-$ as the sums of the value of the ranks of the originally positive and negative observations, respectively. Significance levels are based on the fact that if H_0 is true, then there are $2n$ equally likely ways for the n ranks to receive signs. As test statistic, we use $W = MIN(W-, W+)$. For small samples ($N \leq 30$), the critical regions must be found from some table. For $N > 30$, the test statistic W approaches a normal distribution with a mean of $n(n+1)/4$ and a variance of $n(n+1)(2n+1)/24$. However, to increase speed, we should consider using the t-test when $n > 20$. The t-test to use is the standard one-sample test.

Testing $\mathbf{H}_5$

To test against the alternative H_5, one may use the Kolmogorov test.

Remark

The alternatives are formulated such that a one-sided test may appear most appropriate, except for H_5. This is hardly an important point, however.

UP - F, value in points

Tracks

1. Track 1: unmarked points
2. Track 2: function

Question

In the unmarked points of track 1, is the average value of the function in track 2 smaller/different/larger than expected by chance?

Comment:

- The test is analytic and assumes that the function is white noise. The assumption is also satisfied if the distance between points are so large that there is the correlation between neighbouring points is small.
- We assume the function in track 2 is fixed and that the points in track 1 are independent of the function values in track 2.
- Significance is determined by means of p-values. Small p-values identify bins where the function values are smaller/different/larger than expected in the points of track 1.
- If the points in track 1 depend on other tracks, it is possible to condition the test on an intensity track using this information.

Bins

The genome (or the areas of the genome under study) are divided into small regions, called bins. The tests are performed in each bin.

Hypothesis tested

For each bin i we have one null hypothesis
$\mathbf{H}_0$: *In the unmarked points of track 1, the average value of the function in track 2 is the same as the average function value.*

There are three alternative hypotheses:
$\mathbf{H}_1$: *In the unmarked points of track 1, the average value of the function in track 2 is smaller than the average function value.*
or
$\mathbf{H}_2$: *In the unmarked points of track 1, the average value of the function in track 2 is different than the average function value.*
or
$\mathbf{H}_3$: *In the unmarked points of track 1, the average value of the function in track 2 is larger than the average function value.*

Statistics and rejection of the null hypothesis

Let n be the number of base pairs in the bin, and let Y_i, $i = 1, 2, \cdots, n$, be the function values in the bin. We assume $Y_i \sim N(\mu, \sigma^2)$. Define the average $\bar{Y} = \frac{1}{n}\sum_{i=1}^{n} Y_i$. Also, define $G_i = 1$ if there is a point in track 1 and $G_i = 0$ otherwise. Define $X = \frac{1}{m}\sum_{i=0}^{n} G_i Y_i$ where m is the number of unmarked points in track 1. Notice that under the null hypothesis $EX = E\bar{Y}$ and

$$X - \bar{Y} = \sum_{i=1}^{n} (\frac{G_i}{m} - \frac{1}{n}) Y_i.$$

Define the constant K from the following expression

$$Var(X - \bar{Y}) = \sum_{i=1}^{n} (\frac{G_i}{m} - \frac{1}{n})^2 \sigma^2 = (K\sigma)^2$$

and the sample variance

$$S^2 = \frac{1}{n-1} \sum_{i=1}^{n} (Y_i - \bar{Y})^2.$$

Under the null hypothesis $S^2(n-1)/\sigma^2$ is χ^2-distributed with $n-1$ degrees of freedom. Then the variable

$$T = (X - \bar{Y})/SK$$

is t-distributed with $n - 1$ degrees of freedom. We find the p-value from this distribution depending on the alternative hypothesis.

The test described above is very similar to a standard two sample t-test and the tests will probably give almost identical result. The test described above may be better in reusing previous calculated data and hence reduce CPU time.

Alternative assumption using an intensity track

Assume both the position of the points in track 1 and the function values in track 2 depend on a third track, denoted track 3. We then want to find out if the average value of the function in the points of track 1 is different from the average function values when we also take track 3 into consideration. Track 3 is used for making an intensity track W that gives a weight to each base pair.

For each bin i we have one null hypotheses
$\mathbf{H}_0$: *In the unmarked points of track 1, the average value of the function in track 2 is the same as the weighted average function value.*

There are three alternative hypotheses:
$\mathbf{H}_1$: *In the unmarked points of track 1, the average value of the function in track 2 is smaller than the weighted average function value.*
or
$\mathbf{H}_2$: *In the unmarked points of track 1, the average value of the function in track 2 is different than the weighted average function value.*
or
$\mathbf{H}_3$: *In the unmarked points of track 1, the average value of the function in track 2 is larger*

than the weighted average function value.

We define an intensity track W_i, $i = 1, 2, \cdots, n$, for the points in track 1 conditioned on track 3. W_i is the probability for a point in track 1, conditioned on the value in track 3. We assume that W_i takes k discrete values. Let $q(i)$ be a function that from base pair number i finds the index $1, 2, \cdots, k$ to the discrete value of W_i. Then $W_i = W_j$ if and only if $q(i) = q(j)$. Then we assume $Y_i \sim N(\mu_{q(i)}, \sigma^2)$ where the expectation depends on W_i. Define the variable

$$Z = \frac{1}{\sum_{i=1}^{n} W_i} \sum_{i=1}^{n} W_i Y_i.$$

Notice that $EX = EZ$ and

$$X - Z = \sum_{i=1}^{n} (\frac{G_i}{n} - \frac{W_i}{\sum_{j=0}^{n} W_j}) Y_i.$$

Define the constant K from the following expression

$$Var(X - Z) = \sum_{i=1}^{n} (\frac{G_i}{n} - \frac{W_i}{\sum_{j=0}^{n} W_j})^2 \sigma^2 = (K\sigma)^2.$$

Furthermore, define $\bar{Y}_j$ as the average of Y_i in the base pair where $q(i) = j$ and

$$S^2 = \frac{1}{n-k} \sum_{i=1}^{n} (Y_i - \bar{Y}_{q(i)})^2.$$

Under the the null hypothesis $S^2(n-k)/\sigma^2$ is χ^2-distributed with $n-k$ degrees of freedom. Then the variable

$$T = (X - Z)/SK$$

is t-distributed with $n-k$ degrees of freedom. We find the p-value from this distribution depending on the alternative hypothesis.

There is a similar standard two sample t-test and the tests will probably give almost identical result. The test described above may be better in reusing previous calculated data and hence reduce CPU time.

A slightly better approach is to use a control track. Instead of assuming W_i takes k discrete values and the definition of $q(i)$ above, we may use a control track Q_i taking categorical values. We then assume $Y_i \sim N(\mu_{Q_i}, \sigma^2)$ where the expectation depends on Q_i which is more general than when we use intensity as described above. The only change using a control track instead of an intensity track is the definition of $\bar{Y}_j$ and S^2. Define $\bar{Y}_j$ as the average of Y_i in the base pair where $Q_i = j$ and

$$S^2 = \frac{1}{n-k} \sum_{i=1}^{n} (Y_i - \bar{Y}_{Q_i})^2.$$

US - F, value in segment

Tracks

1. Track 1: unmarked segment
2. Track 2: function

Question

In the unmarked segments of track 1, is the average value of the function in track 2 smaller/different/larger than expected by chance?

Comment:

- We assume the function in track 2 is fixed and that the segments in track 1 are independent of the function values in track 2 under the null hypothesis. The segments in track 1 are preserved and randomized with different algorithms in order to determine whether to reject the hypothesis.
- Significance is determined by means of p-values. Small p-values identify bins where the function values are smaller/different/larger than expected in the segments of track 1.
- The p-values are found by simulation.

Bins

The genome (or the areas of the genome under study) are divided into small regions, called bins. The tests are performed in each bin.

Hypothesis tested

For each bin i we have one null hypothesis
$\mathbf{H}_0$: *In the unmarked segments of track 1, the average value of the function in track 2 is the same as the average function value in the bin.*

There are three alternative hypotheses:
$\mathbf{H}_1$: *In the unmarked segments of track 1, the average value of the function in track 2 is smaller than the average function value in the bin.*
or
$\mathbf{H}_2$: *In the unmarked segments of track 1, the average value of the function in track 2 is different than the average function value in the bin.*
or
$\mathbf{H}_3$: *In the unmarked segments of track 1, the average value of the function in track 2 is larger than the average function value in the bin.*

Statistics and rejection of the null hypothesis

Let X be the average function value in track 2 evaluated in the base pairs inside segments of track 1. The distribution for this test statistics is not know for any of the permutation and randomization of the segments of track 1. It is necessary with MC simulation in order to decide whether to reject the hypothesis.

F - F, similarity (correlation)

Tracks

- Track 1: *Function*
- Track 2: *Function*

Question

Where are the two functions similar/associated/correlated ?

Comment:

- Correlation is measured in different ways.
- The question is answered in the setting of statistical hypothesis testing. We perform the test inside a series of bins of the genome.
- Significance is determined by means of a p-value calculated for each subinterval. Small p-values identify regions with significant results, where the tracks differ.
- The p-values are computed as explained below, where the null hypotheses are explained in detail.

Refined questions

Alternative A1

- Where are the two functions associated?

Alternative A2

- Where are the two functions positively associated?

Alternative A3

- Where are the two functions negatively associated?

Bins

The genome (or the areas of the genome under study) are divided into small regions, called bins. The tests are performed in each bin.

Hypotheses tested

- A1 H_0: No association against H_a: Association
- A2 H_0: No association against H_a: Positive association
- A3 H_0: No association against H_a: Negative association

Tests and test statistics

Similarity of the two functions can be studied in various ways. We focus here on simple tests for correlation like associations between the two series.

Inside a bin, assume we have n observation pairs (x_i, y_i), where x_i is a data point of track 1 in position i and y_i is a data point of track 2 in position i. We wish to test if certain values of x and y have a tendency to occur together, for instance that both track 1 and track 2 tend to have high values or both low values in the same (intervals of) base pairs, which would be a positive association.

The n observation pairs could be the function values in all base pairs inside the bin. But it is likely that each function exhibits (strong, positive) autocorrelation, that is, dependency between function values in neighbouring sites inside each track, f.ex. between x_i and x_j. This will result in too small p-values if ignored, because the following calculations are based on assumptions of n independent observation pairs. To reduce autocorrelation, we divide each bin into n sub bins and use a representative from each sub bin as the n data points for each track in each bin. Such a representative could be the mean, the median or the function value in the midpoint of the sub bin.

Based on these n pairs of observations, we test for linear or non linear but monotone relationships between the two tracks inside each bin. The number of sub bins n should typically be around 20-30. Non smooth functions require more sub bins.

- **Option 1: Pearson correlation** (Assuming binormality and a linear relationship between x and y.)

  Test statistic

  $$T_n = \frac{r_{xy}\sqrt{n-2}}{\sqrt{1-r_{xy}^2}}$$

  where r_{xy} is the empirical correlation coefficient

  $$\frac{\sum x_i y_i - n\bar{x}\bar{y}}{(n-1)s_x s_y}.$$

  Under the null hypothesis of no correlation, T_n has a $t(n-2)$ distribution.

- **Option 2: Spearman correlation** (No assumption on normality, no linear assumption, measures any monotone relationship between x and y.)

  Substitute $x_1, x_2, ..., x_n$ with their ranks, and the same with $y_1, y_2, ..., y_n$. In the case of ties (equal values for two or more measurements), give the same rank to all of the involved values, which should be the mean of the ranks that they otherwise would have had. Calculate r_{xy} above with the observations substituted by their ranks.

  If $n \geq 20$, we use the test statistic T_n and the $t(n-2)$ distribution above to find a p-value. If $n < 20$, precalculated tables for p-values are available.

  If no ties are present, the Spearman r_{xy} can be very easily calculated as

  $$r_{xy} = 1 - \frac{6\sum d_i^2}{n(n^2-1)},$$

where d_i = rank of x_i - rank of y_i.

- **Option 3: Kendall's tau** (Same assumptions as for Spearman, but different measure of association.)

  Among all $n(n-1)/2$ possible pairwise comparisons $\{i, j\}$, let

$$C = \#\text{ pairs where } x_j - x_i \text{ and } y_j - y_i \text{ have the same sign (Concordant)}$$
$$D = \#\text{ pairs where } x_j - x_i \text{ and } y_j - y_i \text{ have the opposite sign (Discordant)}$$

  and

$$\tau = \frac{C - D}{n(n-1)/2},$$

  which is Kendall's tau. In the case of ties ($x_i = x_j$, $y_i = y_j$, or both), τ is instead defined as

$$\tau = \frac{C - D}{\sqrt{[\begin{pmatrix} n \\ 2 \end{pmatrix} - n_x][\begin{pmatrix} n \\ 2 \end{pmatrix} - n_y]}}.$$

  Here n_x and n_y are the number of ties involving x and y, respectively.

  Test statistic is in both cases

$$Z_n = \frac{3\tau\sqrt{n(n-1)}}{\sqrt{2(2n+5)}}$$

  which is $N(0, 1)$ when n is large. Should be OK for for $n \geq 20$. Tabulated p-values are available for n up to 50, but only in the situation of no ties.

MP - MP, similar marks in nearby points

Tracks

1. Track 1: marked points
2. Track 2: marked points

We assume that either the marks in both tracks are categorical or the marks in both tracks are continuous, discrete, ordered categorical and not ordered categorical.

Question

Is the mark of a points in track 1 and the mark of its nearest neighbour point in track 2 independent?

Comment:

- We assume the position of the points in track 1 and the track 2 are fixed. We permutate only the marks of the points in one or both tracks.
- We identify the point in tracks 2 that is the nearest to each point in track 1. There are several different options. Nearest in the direction of lower base pair number, in both directions and in the direction of higher base pair number. It is necessary with a rule of preference if there are points at same distance in both directions. We may neglect neighbours that are further apart than a maximum distance. Some point in track 2 may be the nearest neighbour to several points in track 1 and some points may not be the nearest neighbour to any points in track 1. We assume that this occurs so seldom that it does not dominate the statistics.
- Significance is determined by means of p-values. Small p-values identify bins where the marks of the points in track 1 are not independent of the marks of the points of track 2.
- The p-values are found by an analytic calculation or MC simulation.

Bins

The genome (or the areas of the genome under study) are divided into small regions, called bins. The tests are performed in each bin.

Hypothesis tested

For each bin i we have the null hypothesis
$\mathbf{H}_0$: *The mark of a points in track 1 and the mark of its nearest point in track 2 are independent.*

The alternative hypothesis is:
$\mathbf{H}_1$: *The marks of a points in track 1 depends the mark of its nearest point in track 2.*

Statistics and rejection of the null hypothesis, categorical variables

In this section we assume that the marks of both tracks are categorical variables. Let r be the number of categories for marks of points in track 1 and let c be the number of categories for marks of points in track 2. Furthermore, let $O_{i,j}$ be the number of observations of points from track 1 with mark equal i where its nearest neighbour in track 2 has mark j. In this test we consider these pairs of marks and neglect that some points in track 2 may be part of several pairs and that some points in both tracks may be part of no pairs. The table with the $O_{i,j}$ values is a contingency table with r rows and c columns.

Let N be the total number of pairs, i.e. $N = \sum_{i=1}^{r} \sum_{j=1}^{c} O_{i,j}$. If the marks of the pairs are independent, we expect $O_{i,j} \approx E_{i,j}$ where

$$E_{i,j} = \frac{1}{N} \sum_{k=1}^{r} O_{k,j} \sum_{k=1}^{c} O_{i,k}.$$

Let

$$X = \sum_{i=1}^{r} \sum_{j=1}^{c} \frac{(O_{i,j} - E_{i,j})^2}{E_{i,j}}.$$

Under the null hypothesis X is χ^2-distributed with $(r-1)(c-1)$ degrees of freedom. This is an approximation that is considered accurate if all $O_{i,j} > 10$. (ref. Wikipedia/Pearson's chi-square test). We find the p-value from this distribution. The combinations of i and j that give the largest contribution to the double sum in X, are the cells where the deviation from the independence assumption is largest.

Statistics and rejection of the null hypothesis, continuous or discrete variables

In this section we assume that the marks of both points and segments are continuous or discrete variables. Let X_i be the mark of a point in track 1 and Y_i the mark of its nearest neighbour in track 2, $i = 1, 2, \cdots, n$. We use the following test statistic:

The sample correlation

$$r_{x,y} = \frac{\sum_{i=1}^{n} (X_i - \bar{X}_i)(Y_i - \bar{Y})}{(n-1) s_x s_y} = \frac{\sum_{i=1}^{n} X_i Y_i - n \bar{X} \bar{Y}}{(n-1) s_x s_y},$$

where $\bar{X} = \frac{1}{n} \sum_{i=1}^{n} X_i$, $\bar{Y} = \frac{1}{n} \sum_{i=1}^{n} Y_i$, $s_x^2 = \frac{1}{n-1} \sum_{i=1}^{n} (X_i - \bar{X})^2$, and $s_y^2 = \frac{1}{n-1} \sum_{i=1}^{n} (Y_i - \bar{Y})^2$.

Spearman's rank correlation is defined as the sample correlation except that it uses the ranks x_i and y_i instead of the original data X_i and Y_i.

Kendall τ rank correlation is then defined as

$$\tau = \frac{2(n_c - n_d)}{n(n-1)}.$$

where n_c is the number of concordant pairs i.e. the number of pairs where $(X_i - X_j)(Y_i - Y_j) > 0$ and n_d is the number of of disconcordant pairs i.e. the number of pairs where $(X_i - X_j)(Y_i - Y_j) < 0$. The pairs where both $X_i = X_j$ and $Y_i = Y_j$ are both condordant and disconcordant, but are in fact not critical for the definition of Kendall τ.

The distribution for the sample correlation, Spearman's rank correlation and Kendall τ are known and we may find the p-value from these distributions.

In addition, we may use the test statistics

$$Z_1 = \sum_{i=1}^{n} (X_i - Y_i)^2,$$

and

$$Z_2 = \sum_{i=1}^{n} |X_i - Y_i|$$

The distribution for these test statistics are not known and it is necessary with MC simulations in order to decide whether to reject the hypothesis.

MP - MS, similar marks of points and segments where points are inside segments.

Tracks

1. Track 1: marked points
2. Track 2: marked segments

We assume that either the marks in both tracks are categorical or the marks in both tracks are continuous, discrete, ordered categorical and not ordered categorical.

Question

Are the marks of the points in track 1 that are inside segments of track 2 independent?

Comment:

- We assume the position of the points in track 1 and the entire track 2 are fixed. We permutate only the marks of the points in track 1.
- Significance is determined by means of p-values. Small p-values identify bins where the marks of the points in track 1 are not independent of the marks of the segments of track 2.
- The p-values are found by an analytic calculation or MC simulation.

Bins

The genome (or the areas of the genome under study) are divided into small regions, called bins. The tests are performed in each bin.

Hypothesis tested

For each bin i we have the null hypothesis
$\mathbf{H}_0$: *The marks of the points in track 1 that are inside segments of track 2, are independent of the marks of the segments.*

The alternative hypothesis is:
$\mathbf{H}_1$: *The marks of the points in track 1 that are inside segments of track 2 depend on the marks of the segments.*

Statistics and rejection of the null hypothesis, categorical variables

In this section we assume both points in track 1 and segments of track 2 have categorical marks. Let r be the number of categories for marks in points in track 1 and let c be the number of categories for marks in segments in track 2. Furthermore, let $O_{i,j}$ be the number of observations of points from track 1 with mark equal i that are inside segment from track 2

with mark j. In this test we neglect all points of track 1 that are not inside segments of track 2. The table with the $O_{i,j}$ values is denoted a contingency table with r rows and c columns.

Let N be the total number of points from track 1 that are inside segments in track 2, i.e. $N = \sum_{i=1}^{r}\sum_{j=1}^{c} O_{i,j}$. If the marks of the points are independent of the marks of the segments, we expect $O_{i,j} \approx E_{i,j}$ where

$$E_{i,j} = \frac{1}{N}\sum_{k=1}^{r} O_{k,j}\sum_{k=1}^{c} O_{i,k}.$$

Let

$$X = \sum_{i=1}^{r}\sum_{j=1}^{c}\frac{(O_{i,j}-E_{i,j})^2}{E_{i,j}}.$$

Under the null hypothesis X is χ^2-distributed with $(r-1)(c-1)$ degrees of freedom. This is an approximation that is considered accurate if all $O_{i,j} > 10$. (ref. Wikipedia/Pearson's chi-square test). We find the p-value from this distribution. The combinations of i and j that give the largest contribution to the double sum in X are the cells where the deviation from independence assumptions is largest.

Statistics and rejection of the null hypothesis, continuous or discrete variables

In this section we assume both points in track 1 and segments of track 2 have continuous or discrete marks. Let X_i be the mark of a point in track 1 that is inside a segment in track 2 with mark Y_i for $i = 1, 2, \cdots, n$. We use the following test statistics:

The sample correlation

$$r_{x,y} = \frac{\sum_{i=1}^{n}(X_i-\bar{X}_i)(Y_i-\bar{Y})}{(n-1)s_x s_y} = \frac{\sum_{i=1}^{n} X_i Y_i - n\bar{X}\bar{Y}}{(n-1)s_x s_y}$$

where $\bar{X} = \frac{1}{n}\sum_{i=1}^{n} X_i$ and $\bar{Y} = \frac{1}{n}\sum_{i=1}^{n} Y_i$ and $s_x^2 = \frac{1}{n-1}\sum_{i=1}^{n}(X_i-\bar{X})^2$ and $s_y^2 = \frac{1}{n-1}\sum_{i=1}^{n}(Y_i-\bar{Y})^2$.

Spearman's rank correlation is defined as the sample correlation except that it uses the ranks x_i and y_i instead of the original data X_i and Y_i.

Kendall τ rank correlation is then defined as

$$\tau = \frac{2(n_c-n_d)}{n(n-1)}.$$

where n_c is the number of concordant pairs i.e. the number of pairs where $(X_i - X_j)(Y_i - Y_j) > 0$ and n_d is the number of of disconcordant pairs i.e. the number of pairs where $(X_i - X_j)(Y_i - Y_j) < 0$. The pairs where both $X_i = X_j$ and $Y_i = Y_j$ are both condordant and disconcordant, but are in fact not critical for the definition of Kendall τ.

The distribution for the sample correlation, Spearman's rank correlation and Kendall τ are known and we may find the p-value from these distributions.

In addition, we may use the test statistics

$$Z_1 = \sum_{i=1}^{n}(X_i-Y_i)^2,$$

and

$$Z_2 = \sum_{i=1}^{n} |X_i - Y_i|$$

The distribution for these test statistics are not know and it is necessary with MC simulations in order to decide whether to reject the hypothesis.

UP - MS, Located in highly marked segments

Tracks

- Track 1: Unmarked points
- Track 2: Segments with an attached variable/mark

Remark: Mark of Track 2 assumed to be real numbers or an ordered categorical variable (including the binomial case).

Questions

Is there within the considered bin a correlation between mark values of track 2 and the number of points in the segments of track 1?

Simple model: Segments either of equal length or the segment length is unimportant

We then have a set of pairs (number of points, mark value), and we may use correlation tests. Specific assumptions on the relations between the number of points and the mark value will normally be difficult to establish, and we thus use the well-known non-parametric test based on Kendall's tau. Note that the null hypothesis of no correlation may be rejected either due to some relation between the marks and points or due to factors affecting both, e.g. both number of points and mark values being systematically high in certain areas within the bin.

Alternative model: For fixed mark value, the number of points is approximately proportional to segment length.

Assume that the values of the mark are real numbers, and define:

X_i: Number of points in segment i.

Y_i: Value of variable in segment i.

L_i: Length of segment i.

Choose model (e.g. based on graphical displays):

Model 1: $X_i/L_i = \alpha + \beta Y_i$

Model 2: $\ln(X_i + \epsilon)/L_i = \alpha + \beta Y_i$

Model 3: $\ln(X_i + \epsilon)/L_i = \alpha + \beta \ln Y_i + \epsilon$

The selected model is tested by ordinary regression. Note that extension to more than one mark/variable is simple, as is the use of nominal variables (using general linear models).
