# Supplementary material for: The Genomic HyperBrowser: inferential genomics at the sequence level
Source: Genome Biol. 2010 Dec 23;11(12):R121. doi: 10.1186/gb-2010-11-12-r121 (PMC3046481; doi:10.1186/gb-2010-11-12-r121)
Supplement: Additional file 3 — Supplementary note on simulation. Description of basic algorithms for simulating synthetic tracks, used to assess statistical tests. [file gb-2010-11-12-r121-S3.doc]

**Supplementary note on simulation**

This note describes how to simulate synthetic tracks. The simulation algorithms are the most basic, with few parameters and short simulation algorithms. This may be used in testing different algorithms. We also include how this may be combined with intensity tracks.

1. Simulation of point tracks

Let X_i_ be the distances between the points. X_1_ is the distance from start of track to first point. The simulation algorithm includes clusters.

For i =1,2,3,… until end of track

With probability p, let X_i_ ~expon (λ_A_), and with probability 1-p, let X_i_ ~expon (λ_B_)

expon (λ_A_) means exponentially distributed (i.e. density λ_A_exp (-λ_A_x)) with parameter λ_A_.

There are three parameters:

λ_A_ determines distance between clusters,

λ_B_ determines distance within cluster and

p determines number of points in cluster.

If p=1, all points are independent and expected distance between points is 1/λ_A_ and median ln(2)/ λ_A_.

If p<1, there are clusters. The number of points in the cluster is geometrically distributed. The expected number of points in the cluster is 1/p. Hence, p=1, means one point in each cluster, i.e. all points are independent. The expected distance between points in a cluster is 1/λ_B_ and median ln(2)/ λ_B_. We should have λ_A_ < λ_B_. The expected distance between last point in a cluster and first points in the next cluster is 1/λ_A_.

2. Simulation of segment tracks

Assume overlap between segments

We may use the same algorithm as point tracks where X_i_ is the distance between centre points of segments. If the length of the segment is at the form 2n, we define the centre point to be the n’th base pair in the segment.

The length or the logarithm of the length of each segment may be uniformly distributed in (L_A_, L_B_). This gives 5 parameters

λ_A_ determines distance between clusters,

λ_B_ determines distance within cluster,

p determines number of points in cluster, and

(L_A_, L_B_) specifies the length of the segments.

Assume no-overlap between segments

We may use the same algorithm as above, but now let X_i_ be the distance between the segments. (i.e. from end of one segment to start of the following. )

3. Simulation of function tracks

One level

Let the values of the function track be f_i_ . Assume first only an AR(1) process with the following algorithm:

f_0_ ~ N(a, σ^2^/b)

For i =1,2,3,…. until end of track

f_i_ = a + b (f_i-1_ –a) + ε_i_ where ε_i_ ~ N(0, σ^2^)

This gives values that varies around the level a. Each value is N(a, σ^2^/b). The correlation between neighboring values is b σ^2^.

Several levels

In many cases it is more realistic that the function values vary around several values. We define k states and the function track is an AR(1) process when the track remains within one state.

Let P be a probability matrix where element p_i,j_ is the probability to move from state i to state j, and let p_i_^M^ be the marginal distribution. Theory for Markov chains implies that the vector p^M^ is an eigenvector of P with 1 as an eigenvalue. In practice, we will probably first specify the marginal distribution p^M^. Then we will find a transition matrix P with the given eigenvector and eigenvalue. The expected number of base pairs in a state is 1/(1- p_i,i_). Hence, we will probably choose the diagonal elements in P close to 1.

One alternative for such a matrix is described below. For this matrix there is equal probability for all the other states when leaving a state:

For i =1,2,3,…,n

Set p_i,i_ as a probability slightly less than 1

Set p_i,j_ =(1- p_i,i_ ) p_i_^M^ /(1-p_i_^M^) for j ≠ i.

Let S_i_ be the state for base pair i. The following algorithm describes a function track with several levels.

For i =1,2,3,… until end of track

If (i=1) then <Simulate S_1_ proportional with p^M^>

else <Simulate S_i_ proportional with pS_i-1,.>

Set k=S_i_

If new state g_i-1_ ~ N(a, σ_k_^2^/b_k_) else g_i-1_ = f_i-1_

f_i_ = a_k_ + b_k_ (g_i-1_ –a_k_) + ε_i_ where ε_i_ ~ N(0, σ^2^_k_)

The parameters are

a_k_ mean value in state k

b_k_ determines AR process in state k

σ_k_ determines noise term in state k

P transition matrix

4. Simulation with intensity tracks

Here we will show how to simulate correlated tracks that may be used when testing null models using intensity tracks. This will be illustrated using GC-content and melting function, but the approach is general.

It is well known that both the melting function and the probability for an exon start is related to GC content. The question is whether there is higher correlation between the melting function and exon start than the indirect correlation through the GC content. Or formulated slightly differently, do we know more about the exon start points knowing both the GC content and the melting function than only knowing the GC content.

Let f^GC^ be the function track of smoothed GC content. This is assumed to be data.

Simulate a melting function track f^M^ by

f^M^ = α f^GC^ + (1-α )f^1^

where α is a constant, f^GC^ is assumed known and f^1^ is a “noise” track independent of all the other tracks and simulated by one of the algorithms above.

Simulate the point track T_i_^E^ for exons start correlated by the smoothed GC content by first simulating a new function track f^E^ by

f^E^ = β f^GC^ + γ f^1^

where β and γ are constants, f^GC^ is assumed known and f^1^ is the same noise term as in the melting function. Define a probability p(f_i_^E^) with a parameter f_i_^E^ such that there is an exon start at base pair i with probability p( f_i_^E^), i.e. P(T_i_^E^=1)= p( f_i_^E^). We could have defined separate noise terms in f^M^ and f^E^, but this would not make the model better since we could then replace p( f_i_^E^) with the expected probability when these additional noise terms are not known.

In order to specify this process, it is necessary to specify the functions f^1^, the parameters α, β, and γ, in addition to the probability function p(x). If we assume f_i_^GC^ and f_i_^1^ are independent and normal distributed with variance σ_GC_^2^ and σ_1_^2^ respectively, then the covariance is as follows:

CoV(f_i_^GC^, f_i_^E^) = αβ σ_GC_^2^, CoV(f_i_^M^, f_i_^E^) = αβ σ_GC_^2^+ (1-α) γ σ_1_^2^

From the specification above it is clear that the additional value of knowing f^M^ in addition to f^GC^ is connected to the track γ f^1^.

Quantification of information from function track when evaluating point tracks

We need to quantify the information gained from one or several function tracks (i.e. f^GC^ and f^M^) when evaluating a point track (i.e. T^E^). Then we may quantify the increased information from knowing both the melting function and GC content compared to only the GC content.

As a measure we use the expected values for the probability for a point in the point track T_i_^E^ in the base pairs where there is a point i.e. T_i_^E^ =1. We use the notation E_TiE=1_{P(T_i_^E^=1)}, E _TiE=1_{P(T_i_^E^=1| f_i_^GC^ )}, and E _TiE=1_{P(T_i_^E^=1| f_i_^M^, f_i_^GC^)} for these expected value probabilities. These three expectations are the expected value of an exon start point in the base pairs where there are exon starts, when using no additional information, when using information about GC-content, and when using information about both the GC content and the melting function, respectively. If the melting function f^M^ gives additional information, the last expected value is larger. Notice that we here study each base pair separately. We do not use any spatial information in the model except that both f^GC^_i_ and f^M^_i_ depend on values in several base pairs close to base pair i.
